# Exceptional Performance in Competitive Ski Mountaineering: An Inertial Sensor Case Study

**DOI:** 10.3389/fspor.2022.854614

**Published:** 2022-04-05

**Authors:** Bengt Kayser, Benoit Mariani

**Affiliations:** ^1^Institute of Sport Sciences, University of Lausanne, Lausanne, Switzerland; ^2^Loriaz Consulting, Vallorcine, France

**Keywords:** skimo, endurance, hypoxia, biomechanics, competition, inertial sensors

## Abstract

Organized biannually in the Swiss Alps since 1984, the “Patrouille des Glaciers” (PDG) is one of the most challenging long-distance ski mountaineering (skimo) team competitions in the world. The race begins in Zermatt (1,616 m) and ends in Verbier (1,520 m), covering a total distance of 53 km with a cumulated 4,386 m of ascent and 4,482 m of descent. About 4,800 athletes take part in this competition, in teams of three. We hereby present the performance analysis of the uphill parts of this race of a member (#1) of the winning team in 2018, setting a new race record at 5 h and 35 min, in comparison with two amateur athletes. The athletes were equipped with the Global Navigation Satellite System (GNSS) antenna, a heart rate monitor, and a dedicated multisensor inertial measurement unit (IMU) attached to a ski, which recorded spatial-temporal gait parameters and transition events. The athletes' GNSS and heart rate data were synchronized with the IMU data. Athlete #1 had a baseline VO_2_ max of 80 ml/min/kg, a maximum heart rate of 205 bpm, weighed 69 kg, and had a body mass index (BMI) of 21.3 kg/m^2^. During the race, he carried 6 kg of gear and kept his heart rate constant around 85% of max. Spatiotemporal parameters analysis highlighted his ability to sustain higher power, higher pace, and, thus, higher vertical velocity than the other athletes. He made longer steps by gliding longer at each step and performed less kick turns in a shorter time. He spent only a cumulative 5 min and 30 s during skins on and off transitions. Skimo performance, thus, requires a high aerobic power of which a high fraction can be maintained for a prolonged time. Our results further confirm earlier observations that speed of ascent during endurance skimo competitions is a function of body weight and race gear and vertical energy cost of locomotion, with the latter function of climbing gradient. It is also the first study to provide some reference benchmarks for spatiotemporal parameters of elite and amateur skimo athletes during climbing using real-world data.

## Introduction

Ski mountaineering (skimo) is a winter sport consisting of climbing snow-covered mountains on alpine skis with special ski bindings that allow pivoting the ski boot at the toe while walking up. By attaching hairy skins—nowadays synthetic—under the skis, sliding backward is prevented. On uphill sections, locomotion can be categorized as a type of gait, with the commonly described spatio-temporal parameters such as stride length and cadence. Typically, on steep and narrow terrain, the track zigzags, imposing regular changes in direction with a pivot rotation called kick turn. Occasionally, very steep sections are overcome on foot with the skis on the backpack. For the descent, one takes the skins off and blocks the ski boot heels in the back bindings to allow alpine-style ski turns (Bortolan et al., 2021).

Skimo is becoming increasingly popular as a competitive sport. It was successfully featured on the 2020 Youth Olympic Winter Games in Lausanne and will be on the program of the Milano-Cortina Winter Olympics in 2026. An International Ski Mountaineering Federation (ISMF) coordinates an increasing number of races to rank athletes and teams during European and world championships. Several disciplines exist such as sprint, vertical, and endurance of which the latter is the oldest. Recent advances in technology now allow field measurements to be combined with laboratory-based measurements (Fasel et al., 2016; Praz et al., 2016a,b; Gellaerts et al., 2018). Nevertheless, there is a relative paucity of research on skimo (Bortolan et al., 2021).

**Figure 1:**
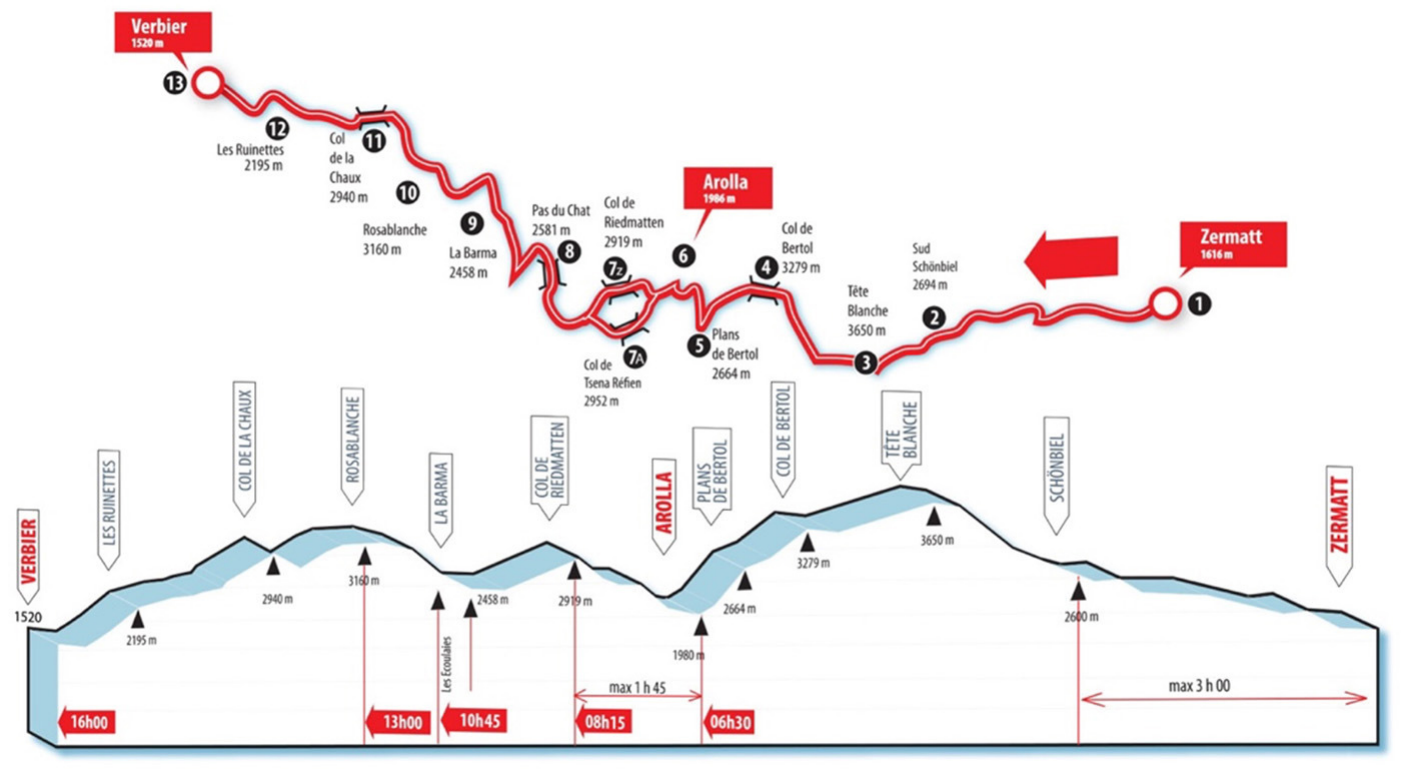
Altitude profile [from the right (East) to the left (West)], with the top part corresponding to the geographical itinerary (reproduced with permission, www.pdg.ch).

The “*Patrouille des Glaciers*” (PDG) (https://www.pdg.ch) is a famous long-distance skimo team event in the Swiss Alps, which gathers a large number of participants (capped at 4,800 in 2018). It is organized every second year, with the next edition scheduled for April 2022 because of the coronavirus disease 2019 (COVID-19) pandemic. There are two routes: the main race from Zermatt (1,616 m) to Verbier (1,529 m) and a shorter one from Arolla (1,986 m) to Verbier. The former covers 53 km with > 4,000 m cumulated ascents and descents between the lowest and highest points, at 1,520 and 3,650 m, respectively (see Figure 1). Athletes in patrols of three compete against each other in various age, civil, and military categories. One patrol member can abandon, but only patrols that arrive complete are ranked. The event gathers participants from a wide range of levels from professional to amateur. The PDG takes place in the second half of April when the days become longer. The race starts at night time in Zermatt with slower teams starting first. The fastest teams then start in the middle of the night to be able to descend at dawn to the village of Arolla, located about midway, to then continue toward the finish in Verbier in daylight.

From 1984 to 2018, the PDG race record has steadily progressed (from 7 h 59 min to 5 h 35 min, respectively). This improvement in performance is likely the result of a combination of advances in technology (skis, bindings, and boots), the weight of gear carried, but also of training modalities and skiing techniques (Bortolan et al., 2021). Praz et al. (2014) reported data collected on 28 participants to an earlier edition of the PDG and found that race performance varied as a function of age, aerobic capacity, the energy cost of locomotion, body fat percentage, and body weight together with the added weight of gear. Based on further field measurements and simulated uphill skimo on a treadmill in a laboratory environment, they found that there may be an optimal combination of high speed and steep slope, where the vertical energy cost is the lowest, provided the aerobic power needed can be maintained by the individual (Praz et al., 2016a,b).

In this case report, we present data collected on an exceptional athlete (#1) who in 2018, together with his two team members, set a new record (5 h 35 min) on the PDG. We confront the data collected on this elite athlete to the literature and to the data from two other participating amateur athletes, whose teams ranked 83 and 194, respectively, among the 345 finishing teams on the same race that day.

## Methods

Demographic information, baseline exercise testing results, and race data were provided voluntarily by the athletes, with the explicit written permission to use the data for research purposes and publication of the results, while the race results were publicly available. Therefore, ethical approval was not necessary because no experiments were performed by the authors.

Routine testing for training purposes took place in the training phase prior to the race using indirect calorimetry with calibrated gas exchange measurement devices during incremental running on treadmills until exhaustion in different laboratories independent of the authors' laboratory. Body mass and stature were measured with standard laboratory apparatus.

**Figure 2:**
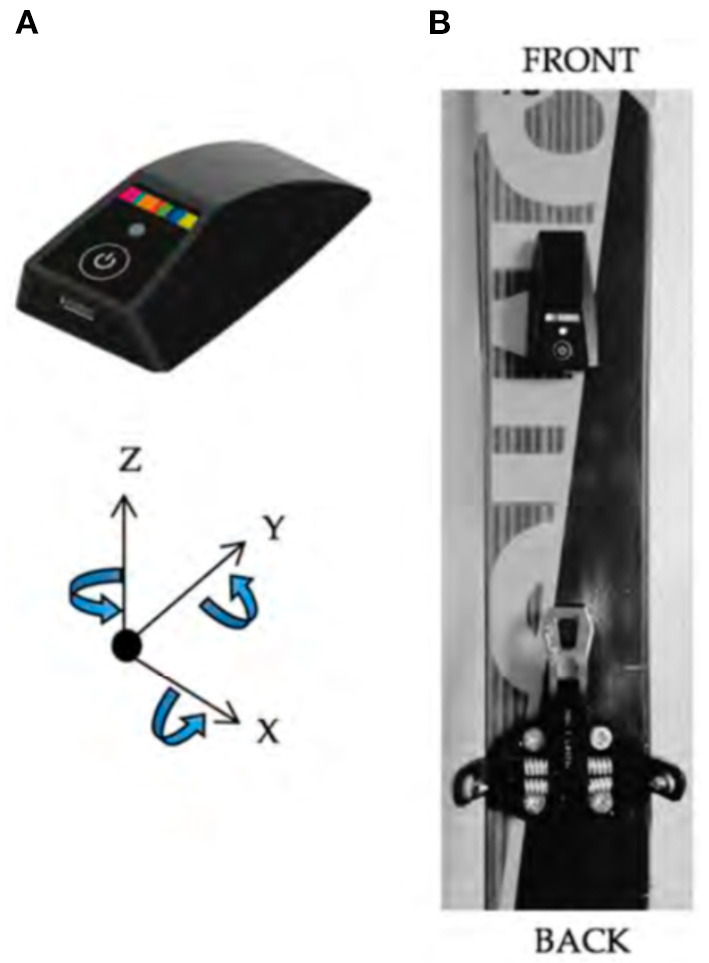
**(A)** Ski-mounted multisensor IMU used by the three athletes (containing a 3D accelerometer, a 3D gyroscope, a barometer, a thermometer, a CPU, and solid-state memory). **(B)** Position on the ski in front of the front-binding (Gellaerts et al., 2018).

During the race, athlete #1 wore his own heart rate/Global Navigation Satellite System (GNSS) monitor and all three were equipped with a single ski-mounted dedicated multisensor inertial measurement unit (IMU) (Figure 2) (Pomocup, Pomoca SA, Switzerland). This IMU allows real-time parameter estimation (cadence, distance from strides, stride duration, stride length, number of strides, slope gradient, and mechanical power) as well as transition detection (kick turns, skin-on, skin-off, ski-on, and off backpack) in order to classify between the different types of locomotion (skimo, walking, and skiing). This IMU was validated with data obtained in 16 skimo athletes who performed different skimo tracks with their own equipment instrumented with the ski-mounted IMU. The results obtained by the algorithm showed precise results with a relative error of <5% on all the parameters (Gellaerts et al., 2018).

The data from the different sensors were synchronized to the race profile according to position time series and exported in a .csv format for analysis. All the calculated parameters were compared relative to athlete #1. The 3 main uphill sections (Tête Blanche, Col de Riedmatten, and Rosablanche, see Figure 1) were subdivided into four subsections of similar altitude differences each, and the means ± SD of the different sensor derived parameters for these sections were calculated.

For athlete #1, we used heart rate to estimate oxygen uptake during the race, assuming a linear relationship at the high heart rates reached during endurance exercise. We corrected for the effect of altitude on VO_2_ max using the following equation: %VO_2_ max = −0.004871 × altitude (m) + 100, derived from published data on trained participants of the study by Mollard et al. (2007). We corrected maximum heart rate (HRmax) as a function of altitude according to Mourot (2018): HRmax (bpm) = −0.0024 × altitude (m) + 0.73, and expressed heart rate as a % of altitude-corrected HRmax. Gross efficiency was calculated as the ratio between mechanical power as estimated with the inertial sensor device and metabolic rate from altitude-corrected estimated VO_2_, assuming an energy equivalent of 20.9 kJ per l O_2_, corresponding to a respiratory exchange ratio of 0.96. The energy cost for vertical displacement was calculated as the energy expended—from altitude-corrected estimated VO_2_—for each vertical meter, expressed in J/kg (normalization with the sum of body mass and gear carried).

## Results

### Anthropometry and Peak Oxygen Uptake

General baseline information on the three male athletes at the time of the race in 2018 is given in Table 1. The low altitude measured aerobic capacity of athlete #1 was 80 ml/min/kg. With the added weight of the skis, boots, clothes, and carried gear (~6 kg) it was reduced to 74 ml/min/kg. Athletes #2 and #3 had, respectively, 18 and 28% less aerobic capacity as compared with #1.

**Table 1:** Athlete characteristics.

	**#1**	**#2**	**#3**
Age	28	29	38
Height (cm)	180	178	185
Weight (kg)	69	77	75
BMI (kg/m^2^)	21.3	24.3	21.9
VO_2_max (ml/min/kg)^*^	80	65	57
HRmax (bpm)^*^	205	182	197

*HRmax: maximum heart rate*.

* *Low altitude incremental treadmill running test to exhaustion results at baseline*.

### Race Times

Intermediate times of the winning team, partitioned along the different uphill and downhill sections of the race are shown in Table 2, with for athletes #2 and #3 their respective percentages of slower performance. On average #1 was 36 and 53% faster than #2 and #3, respectively (Table 2).

**Table 2:** Race times.

	**#1**				**#2**	**#3**
	**Race time**	**Stage time**	**Altitude changes**	**Average vertical speed**	**Relative vertical speed decrease compared to #1**	
	**(hr:min:s)**	**(hr:min:s)**	**(m)**	**(m/hr)**	**(%)**	
Zermatt	00:00:00	00:00:00	-	-	-	-
Schönbiel	01:15:24	01:15:24	984	783	27	42
Tête Blanche	02:18:08	01:02:44	1,050	1,004	32	54
Col de Bertol	02:34:21	00:16:13	−371	−1,373	46	53
Plans de Bertol	02:40:07	00:05:46	−615	−6,399	47	65
Arolla	02:48:57	00:08:50	−684	−4,646	34	50
Col de Riedmatten	03:34:49	00:45:52	939	1,228	34	60
Pas du Chat	03:47:13	00:12:24	−338	−1,635	47	63
La Barma	04:13:13	00:26:00	0	11.3^*^	46	68
Rosablanche	04:57:47	00:44:34	579	780	37	57
Col de la Chaux	05:18:24	00:20:37	−220	−640	35	47
Les Ruinettes	05:27:22	00:08:58	−745	−4,985	28	36
Verbier	05:35:27	00:08:05	−675	−5,010	17	38
Mean ± SD					36 ± 9	53 ± 10

*Speed data of athlete #1 along the various sections of the race. Vertical speed sign indicates uphill and downhill sections in m/h, with the exception of*

* *: horizontal speed in km/hr on the horizontal part of the race along the shores of the Dix Lake, where the strongest athletes adopt a cross-country skating locomotion technique*.

### Performance on the Three Main Climbs

In Table 3, the data of athlete #1, obtained with the ski-mounted IMU and with the heart rate monitor, are presented as means for the three main climbs, partitioned in 4 sections of similar altitude differences each to compare different altitudes, and excluding the sections where the skis were carried on the backpack. On average #1 kept his HR constant around ~86% of his altitude corrected HRmax all along with the climbs.

**Table 3:** Detailed results for athlete #1 during the three main climbs.

**Tête Blanche**	**1,600–2,100 m**		**2,100–2,600 m**		**2,600–3,100 m**		**3,100–3,600 m**	
	**Mean**	**SD**	**Mean**	**SD**	**Mean**	**SD**	**Mean**	**SD**
Power (W)	321	99	263	88	277	94	262	85
Glide (%)	57	10	58	10	49	4	48	2
Ski angle (°)	4	9	2	8	15	4	14	3
Step duration (s)	0.5	0.1	0.5	0.1	0.6	0.1	0.6	0.0
Step length (cm)	99	18	99	18	78	10	81	6
Cadence (spm)	120	19	119	19	102	10	100	6
Heartrate (bpm)	176	21	149	25	173	5	174	5
GE (%)	15	9	12	8	17	6	15	7
Cvert (J/kg/m)	98	43	95	53	61	9	64	19
Fcmax (%)	87	11	74	12	86	3	86	2
VO_2_ (ml/kg/min)^*^	60	7	49	8	56	2	55	2
Vertical speed (m/h)	849	366	661	400	839	418	1,070	239
**Col de Riedmatten**	**2,025–2,225 m**		**2,225–2,425 m**		**2,425–2,625 m**		**2,625–2,825 m**	
Power (W)	299	98	274	78	244	100	257	88
Glide (%)	47	2	50	6	54	6	51	5
Ski angle (°)	18	3	12	6	9	5	12	4
Step duration (s)	0.6	0.1	0.6	0.1	0.5	0.0	0.6	0.0
Step length (cm)	76	5	86	14	96	10	87	8
Cadence (spm)	96	7	105	11	110	6	107	6
Heartrate (pm)	173	4	172	7	171	7	174	5
GE (%)	17	6	16	5	14	6	15	6
Cvert (J/kg/m)	59	5	71	18	82	24	71	14
Fcmax (%)	85	4	86	7	84	7	86	5
VO_2_ (ml/kg/min)^*^	59	1	57	3	56	2	57	2
Vertical speed (m/h)	1,243	100	1,071	216	916	202	1,032	175
**Rosablanche**	**2,414–2,564 m**		**2,564–2,714 m**		**2,714–2,864 m**		**2,864–2,997 m**	
Power (W)	240	71	262	74	237	69	259	41
Glide (%)	51	6	47	6	48	8	45	1
Ski angle (°)	11	5	15	7	14	6	17	2
Step duration (s)	0.6	0.0	0.6	0.1	0.6	0.1	0.7	0.0
Step length (cm)	93	12	83	11	83	16	77	4
Cadence (spm)	105	9	93	10	95	11	89	4
Heartrate (bpm)	173	3	175	5	177	4	176	1
GE (%)	14	4	15	6	14	5	15	4
Cvert (J/kg/m)	89	30	73	31	81	40	62	4
Fcmax (%)	86	2	86	2	87	2	87	1
VO_2_ (ml/kg/min)^*^	57	1	57	2	58	1	57	0
Vertical speed (m/h)	852	244	977	369	990	260	1,164	70

* *VO_2_ corrected for altitude*.

### Comparison Between Athletes

In Table 4 the comparison between the three athletes is shown, of the main parameters derived from the ski-mounted IMU, on the three main climbs and the two minor climbs (excluding the two short sections where the skis were carried on the backpack). On average #1 developed 3.1 Watt/kg while climbing on his skis, taking into account the extra weight carried. While athlete #2 and #3 developed 2.0 and 1.4 Watt/kg, respectively. The main other difference is the higher cadence of athlete #1, who was able to keep it around 100 spm, while athletes #2 and #3 had consistently lower step rates which dropped further over time. Also, glide time in % of step duration was greater in athlete #1 as compared to athletes #2 and #3.

**Table 4:** Spatiotemporal parameters for athlete #1 and amateur athletes #2 and #3 in the five uphill parts of the race.

	**Tête blanche**			**Col de Bertol**			**Col de Riedmatten**			**Rosablanche**			**Col de la Chaux**		
**Athlete:**	**#1**	**#2**	**#3**	**#1**	**#2**	**#3**	**#1**	**#2**	**#3**	**#1**	**#2**	**#3**	**#1**	**#2**	**#3**
Power (W/kg)	3.6	2.3	1.7	2.5	1.6	1.1	3.5	2.4	1.8	3.2	1.9	1.4	2.9	1.8	1.2
Glide (%)	54	51	48	61	57	49	51	47	44	48	45	43	52	51	46
Step duration (s)	0.53	0.64	0.74	0.52	0.58	0.75	0.57	0.68	0.79	0.62	0.73	0.89	0.55	0.68	0.84
Step length (cm)	90	87	88	103	89	88	87	79	81	84	75	79	92	84	84
Cadence (spm)	111	94	82	114	102	81	105	89	77	96	80	69	104	89	72

*Tête Blanche, Col de Riedmatten and Rosablanche are the main uphill sections of the race; Col de Bertol and Col de la Chaux are short uphill sections (see Figure 1). spm: strides per minute*.

**Figure 3:**
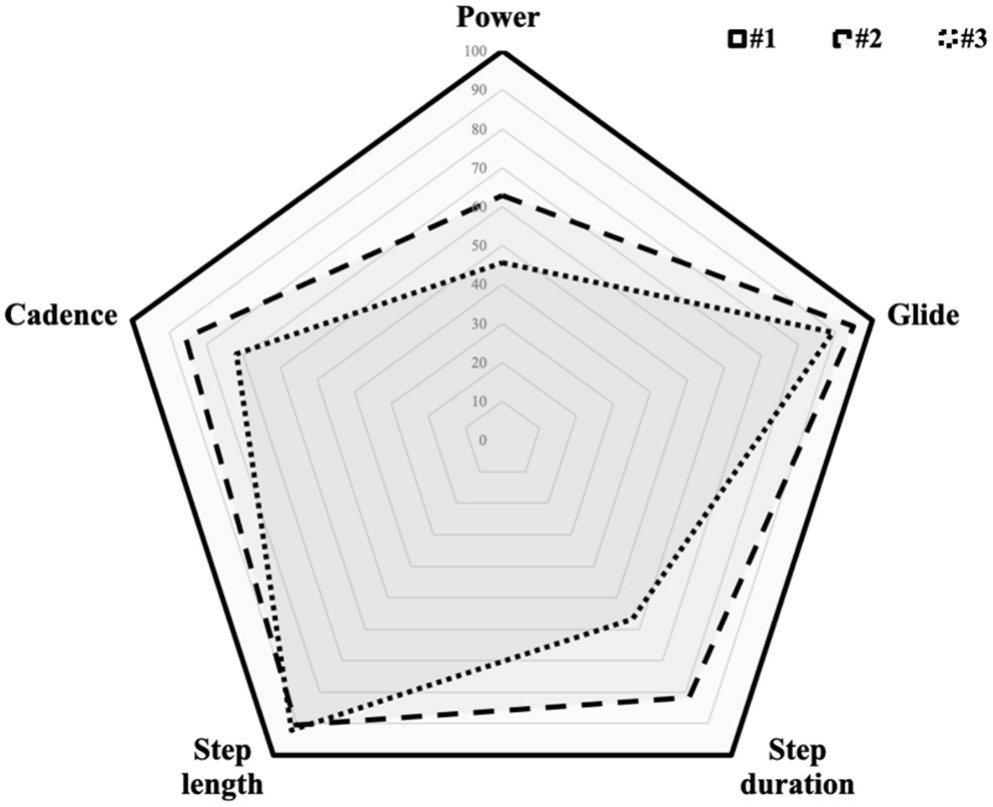
Radar plot for athletes #1 (solid line), #2 (dashed line), and #3 (dotted line).

Thus, spatiotemporal parameter analysis highlighted a higher power, a higher pace, and a higher vertical velocity of athlete #1 compared to athletes #2 and #3. Also stride length was higher for athlete #1 with 91 cm compared to #2 and 3# with 83 and 84 cm on average, respectively. The difference was mainly higher in the second uphill section which was the least steep uphill section of the race. Athlete #1 only spent 5 min 30 s during transitions putting his skins on and off; athletes #2 and #3 spent, respectively, 23 and 45 min. In Figure 3 are shown for comparison and expressed in % of athlete #1, power, glide, step duration, length, and cadence of athletes #2 and #3.

## Discussion

We hereby present observations on three skimo athletes from different teams, including an athlete whose team established in 2018 a new race record of 5 h 35 min on a famous long-distance skimo team event in the Swiss Alps, the “*Patrouille des Glaciers*.” The main findings align with earlier work on factors limiting performance in skimo endurance events, namely, that it takes a high aerobic power of which a high fraction can be maintained for a prolonged time, low body mass, and modern lightweight equipment to climb fast (Bortolan et al., 2021). Praz et al. (2014) analyzed data obtained on 28 participants on a previous PDG and found that age, body weight and weight of carried gear, baseline VO_2_max, and energy cost of locomotion were significantly correlated with performance. Hereunder the results obtained in athlete #1 are confronted to the literature and to the data obtained in athletes #2 and #3.

### Aerobic Power and Heart Rate

Athlete #1 had a baseline peak oxygen uptake during incremental running on a treadmill until exhaustion of 80 ml/min/kg, which can be considered rather good and comparable to that reported for endurance athletes, but not exceptional. A major determinant of endurance performance is not only peak oxygen uptake, but also the fraction that can be maintained for a prolonged time (Maunder et al., 2021). Athlete #1 was able to maintain 85% of his altitude adjusted HRmax, and presumably also a large fraction of his altitude adjusted aerobic power, throughout the race, even though some heart rate drift probably occurred (Mattson et al., 2011). During shorter skimo solo races, heart rates can reach an average of 93% of HRmax, while in the longer team races on average 87% of HRmax is reached (Bortolan et al., 2021). During the climbs, athlete #1 kept his heart rate on average at about 86% of his HRmax, comparable to the values reported in the literature, suggesting that he may have had very limited reserve left. Of note, the differences in aerobic fitness and endurance capacity between members of a team should ideally be very small, since a team is limited by the slowest member, while the team is obliged to progress and finish together.

Assuming a 20% efficiency and taking into account the added weight of clothes, boots, skis, and other gear (6 kg) athlete #1's peak oxygen uptake at low altitude would have allowed him to develop a theoretical peak aerobic mechanical power output of ~5.1 Watts/kg, within the range reported in the literature on endurance athletes, for example, elite cyclists (Lee et al., 2002). The mechanical power developed during the race, estimated with the IMU attached to one of the skis was lower, likely explained by the decrease in maximum sustainable power output as a function of altitude, and the submaximal metabolic rate maintained throughout the race, as indicated by the 85% of HRmax.

### Vertical Speed and Energy Cost of Locomotion

**Figure 4:**
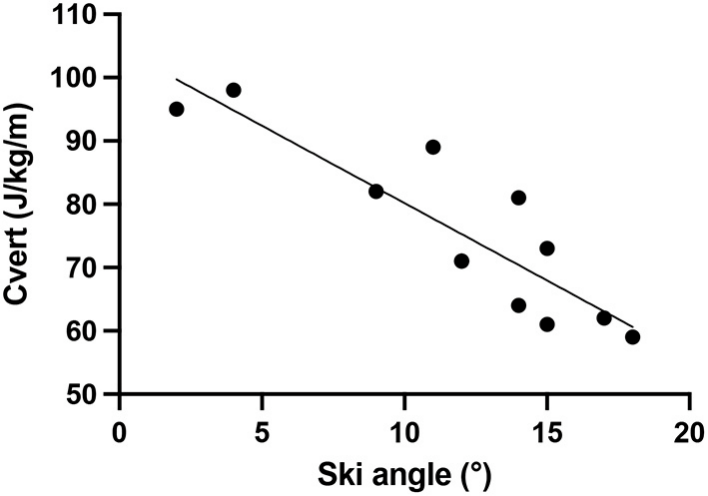
Mean vertical energy cost for the various sections of the 3 main climbs on the PDG as a function of the slope angle for athlete #1. *R*^2^ = 0.78, *P* < 0.0001.

On the uphill parts of skimo races, the objective is to reach the highest point in the shortest time possible. Hence, the vertical energy cost of locomotion is an important determinant of performance. Praz et al. (2016a,b) reported that a combination of high speed and steep slope allows keeping vertical energy cost low, provided the aerobic power needed can be maintained by the individual. A practical problem is that the athlete may choose a suboptimal existing track since there is a trade-off to be made between choosing an energetically more optimal steeper angle and the extra metabolic cost of making a new track in soft snow. On the PDG athletes generally choose existing tracks at a given angle. Presumably, athlete #1's team chose pragmatically to climb along such existing tracks, selecting those that allowed climbing at steeper angles, thus limiting the number of time-consuming kick turns. The final choice of the itinerary was therefore likely the result of constraints imposed by the terrain and any existing tracks, with a range of angles used to progress upwards. This allowed us to investigate the relationship between vertical energy cost and ski angle as shown in Figure 4, showing the mean vertical energy cost for the various sections of the 3 main climbs on the PDG as a function of the ski angle of athlete #1. Steeper angles were accompanied by a significantly lower vertical energy cost. As reported in the Praz et al. studies (2016a; 2016b), it would, thus, seem that there might exist a steeper slope where the vertical energy cost would be even lower.

### Stride Characteristics

The stride characteristics of athlete #1 were distinctly different from those of athletes #2 and #3. The differences observed on all parameters were larger than the maximum IMU's error and can thus be considered as significant. Athlete #1 was able to keep his gliding ratio around 50%, which suggests a locomotory pattern more like a grounded running pattern than walking, which may have optimized the energetic cost of locomotion at a given speed as compared to that of a walking-like locomotory pattern (Davis et al., 2020). Athlete #1 also had and maintained a higher cadence of steps that were longer as compared to athletes #2 and #3. It is of note that athlete #1 achieved faster uphill speed than #2 with both a higher cadence and a greater stride length, while athlete #2 achieved faster uphill speed than #3 mostly by having greater stride length but comparable cadence.

### Transitions and Overall Race Performance

On average athletes #2 and #3 needed 36%, respectively, 53% more time to reach the finish line in Verbier (Table 2). The differences were similar during ascent (33 and 54%, respectively) and descent (34 and 49%, respectively). These differences are likely partly explained by the higher aerobic capacity of athlete #1, but also his better training status and better technique as an elite athlete who entered the competition with the aim to win, or at least to finish as high as possible in the ranking, while athletes #2 and #3 did not participate to win but rather to finish. Indicative of this is the longer time spent by athletes #2 and #3 during the transition phases for taking off the skins for the descents and for putting them back under the skis for the climbs. The winning team only spent a total of 5 min 30 s putting their skins on and off while the teams of athletes #2 and #3 spent 23 and 45 min, respectively. The longer times in the transition phases of the teams of athletes #2 and #3 were likely also spent to get some rest and to drink and eat.

## Limitations

Several limitations should be considered. First, as a case study, the results cannot be generalized and there is a need for further investigation. Second, athlete #2 did not wear a heart rate monitor and we, therefore, could not compare estimates of aerobic power during the race. Third, no other parameters such as saturation were available. Fourth, we could not obtain quality information on the training practices of the three athletes.

## Conclusion and Perspectives

This case report on a member of the winning team on an endurance team skimo event confirms that skimo performance requires a high aerobic power of which a high fraction can be maintained for a prolonged time. Our results further confirm earlier observations that speed of ascent during endurance skimo competitions is the function of body weight and race gear, and vertical energy cost of locomotion, with the latter function of climbing gradient. Now that increasing numbers of athletes routinely record their heart rate, GNSS, and IMU data, as well as other parameters, during both training and competition, on various online platforms, it will become possible to analyze race data of greater numbers and to do more fine-grained analysis, for example, with teams.

## Data Availability Statement

The raw data supporting the conclusions of this article will be made available by the authors, without undue reservation.

## Ethics Statement

Ethical review and approval was not required for the study on human participants in accordance with the local legislation and institutional requirements. The patients/participants provided their written informed consent to participate in this study. Written informed consent was obtained from the individual(s) for the publication of any potentially identifiable images or data included in this article.

## Author Contributions

BM approached BK with the idea for the case report. BK did data extraction and data analysis. The first draft of the manuscript was written by BK. Both authors contributed to the final version.

## Funding

Open access funding was provided by the University of Lausanne.

## Conflict of Interest

BM is employed by Loriaz Consulting SAS. The remaining author declares that the research was conducted in the absence of any commercial or financial relationships that could be construed as a potential conflict of interest.

## Publisher's Note

All claims expressed in this article are solely those of the authors and do not necessarily represent those of their affiliated organizations, or those of the publisher, the editors and the reviewers. Any product that may be evaluated in this article, or claim that may be made by its manufacturer, is not guaranteed or endorsed by the publisher.
