# Erratum: Exceptional performance in competitive ski mountaineering: An inertial sensor case study

**DOI:** 10.3389/fspor.2023.1186995

**Published:** 2023-04-03

**Authors:** Frontiers Production Office

**Affiliations:** Frontiers Media SA, Lausanne, Switzerland

**Keywords:** skimo, endurance, hypoxia, biomechanics, competition, inertial sensors

An Erratum on Exceptional performance in competitive ski mountaineering: an inertial sensor case study By Kayser B and Mariani B. (2022) Front. Sports Act. Living 4:854614. doi: 10.3389/fspor.2022.854614

An omission to the funding section of the original article was made in error. The following sentence has been added: “Open access funding was provided by the University of Lausanne”.

The original version of this article has been updated.

